# Meta-Analytic Comparison of Global RNA Transcriptomes of Acute and Chronic Myeloid Leukemia Cells Reveals Novel Gene Candidates Governing Myeloid Malignancies

**DOI:** 10.3390/cancers14194681

**Published:** 2022-09-26

**Authors:** Staša Jurgec, Gregor Jezernik, Mario Gorenjak, Tomaž Büdefeld, Uroš Potočnik

**Affiliations:** 1 Center for Human Genetics and Pharmacogenomics, Faculty of Medicine, University of Maribor, Taborska ulica 8, 2000 Maribor, Slovenia; 2 Laboratory for Biochemistry, Molecular Biology and Genomics, Faculty of Chemistry and Chemical Engineering, University of Maribor, Smetanova ulica 17, 2000 Maribor, Slovenia; 3 Department for Science and Research, University Medical Centre Maribor, Ljubljanska ulica 5, 2000 Maribor, Slovenia

**Keywords:** AML, CML, meta-analysis, lincRNA, spliceosome

## Abstract

**Simple Summary:**

Despite advances in the understanding of genetic risk factors and molecular mechanisms underlying acute myeloid leukemia (AML) and chronic myeloid leukemia (CML), clinical outcomes of current therapies in terms of disease relapse and mortality rate pose a great economic and social burden. To overcome this, the identification of new molecular prognostic biomarkers and pharmacological targets is crucial. Recent studies have suggested that AML and CML may share common pathogenic mechanisms and cellular substrates. To this end, in the present study, global transcriptome profiles of AML and CML at the molecular and cellular level were directly compared using a combination of meta-analysis and modern statistics, and novel candidate genes and specific biological processes associated with the pathogenesis of AML and CML were characterized. Our study significantly improves our current understanding of myeloid leukemia and will help develop new therapeutic targets and biomarkers for disease progression, management and treatment response.

**Abstract:**

Background: Acute myeloid leukemia (AML) and chronic myeloid leukemia (CML) represent a group of hematological malignancies characterized by the pathogenic clonal expansion of leukemic myeloid cells. The diagnosis and clinical outcome of AML and CML are complicated by genetic heterogeneity of disease; therefore, the identification of novel molecular biomarkers and pharmacological targets is of paramount importance. Methods: RNA-seq-based transcriptome data from a total of five studies were extracted from NCBI GEO repository and subjected to an in-depth bioinformatics analysis to identify differentially expressed genes (DEGs) between AML and CML. A systemic literature survey and functional gene ontology (GO) enrichment analysis were performed for the top 100 DEGs to identify novel candidate genes and biological processes associated with AML and CML. Results: LINC01554, PTMAP12, LOC644936, RPS27AP20 and FAM133CP were identified as novel risk genes for AML and CML. GO enrichment analysis showed that DEGs were significantly associated with pre-RNA splicing, reactive oxygen species and glycoprotein metabolism, the cellular endomembrane system, neutrophil migration and antimicrobial immune response. Conclusions: Our study revealed novel biomarkers and specific biological processes associated with AML and CML. Further studies are required to evaluate their value as molecular targets for managing and treating the myeloid malignancies.

## 1. Introduction

Myeloid leukemias are a group of hematological cancers that affect cells of the myeloid lineage. Myeloid leukemias can be divided into acute and chronic types of disease based on genetic, immunophenotypic, morphological, pathophysiological and clinical features. Chronic myeloid leukemia (CML) is characterized by leukocytosis and an accumulation of granulocytes and their precursors. A progression of CML is biphasic or triphasic; an initial indolent chronic phase is followed by an accelerated phase or a blast phase or both. A reciprocal chromosomal translocation t(9;22) resulting in the Philadelphia chromosome is found in 90 to 95% of all CML cases; however, other chromosomal abnormalities and gene mutations are also associated with CML. Acute myeloid leukemia (AML) is characterized by the clonal expansion of myeloid blasts in the peripheral blood, bone marrow or other tissues, and it can involve a single or all myeloid lineages. AML is a rapidly progressing disease due to clonal expansion of myeloid blasts. Genetic abnormalities associated with AML are more heterogeneous than in CML comprising at least 24 different genetic subgroups [1,2].

Despite the differences, AML and CML may share common pathogenic mechanisms. It is well-established that a fast-progressing blast phase of CML resembles the progression of AML. Moreover, it has been suggested very recently that both AML and CML arise from leukemia stem cells harboring unique genetic abnormalities [3] suggesting to some extent a common origin of diseases. According to this rather simplified model, leukemic stem cells that give rise to blast phase CML and AML originate from hematopoietic stem cells and lineage-restricted progenitor cells. Similarly, leukemia stem cells in CML arise from hematogenic stem cells or in even more primitive hemangioblasts. The effect of genes and somatic mutations on the transformation of myelodysplastic syndrome into secondary AML was further confirmed in an induced pluripotent stem cell model in vitro, using sequential CRISPR gene editing [4]. This led us to hypothesize that comparing gene expression profiles of AML and CML, two endpoints of leukemia affecting the myeloid lineage, would lead to the discovery of molecular marker that are essential in the chronic and acute forms of the disease and contribute to further our understanding of myeloid neoplasms. In the present study, we performed a comparative meta-analysis of the global transcriptome between AML and CML. We characterized five new differentially expressed candidate genes between AML and CML. Gene ontology (GO) annotations further suggested a role of U2-dependent pre-RNA splicing, RNA, reactive oxygen species and glycoprotein metabolism, the cellular endomembrane system, secretory granules, neutrophil migration, antimicrobial humoral immune response and antifungal defense system in the pathophysiology of AML and CML.

## 2. Materials and Methods

### 2.1. Search Strategy

A systematic search of the NCBI Gene Expression Omnibus (GEO) repository of curated gene expression datasets (https://www.ncbi.nlm.nih.gov/gds/) (accessed on 9 May 2022) was performed using the keyword “myeloid leukemia”. A total of 4104 relevant data series were identified. Subsequently, data series were excluded if the samples used in the experiment were not of human origin (*n* = 604) and if the data reported were not high-throughput sequencing profiling (*n* = 2267). Data series were further excluded if experiments were done on cell lines (*n* = 1090). In addition, data series were excluded if experiments were done on specific blood cell populations, bone marrow biopsies, genetically and pharmacologically manipulated human samples and myeloproliferative disorders that were not defined as chronic myeloid leukemia as well as if data reported were single-cell RNA transcriptomics (scRNA-seq). Of the remaining 15 studies, 5 studies containing publicly available data of global transcriptome analysis (RNA-seq) of blood cells by paired-end tag sequencing were included in the meta-analysis (Table 1).

### 2.2. Data Extraction, Processing and Analysis

Data were downloaded using sratoolkit [11] and technical replicates were combined under one sample identifier. Data analysis was performed using R 4.0.2 environment [12] as described previously [13,14]. Paired-end reads were mapped to the hg19 reference genome and assigned to genomic features using Rsubread 2.2.4 R package and featureCounts [15,16]. Low expressed genes were filtered out based on CPMs corresponding to read counts of 10 and retained genes were normalized using the trimmed mean of M values method (TMM) [17]. Subsequently, mean–variance modeling at the observational level transformation (VOOM) was applied [18]. Differential expression of acute myeloid leukemia relative to chronic myeloid leukemia was determined using linear models and empirical bayes implemented in limma 3.44.3 R package [19] using deconvoluted peripheral blood mononuclear cell composition data. Deconvolution was used to obtain an estimation of the abundances of member cell types in a mixed cell population, using gene expression data in terms of proportions of different white cell subtypes in PBMCs [20]. Using raw counts, transcripts per million (TPM) were calculated, zero values were removed, and the data were deconvoluted using CIBERSORT [20] and LM22 [20] signature matrix. Sample composition in terms of proportions of lymphocytes, monocytes/macrophages and neutrophils was used as a covariate in the model. Zero estimated cell proportions were excluded from analyses. In addition, gene expression results were adjusted for sex. Differential expression was considered for genes with adjusted *q*-value < 0.05 (*p*-value after FDR correction) and loget >2 or <−2 (loget = log2(CML/AML)). In total, three analysis were performed; AML vs. CML, healthy vs. AML and healthy vs. CML. A systematic search of the PubMed literature database and BioGRID ORCS repository was performed for the top 100 DEGs to identify genes that have not yet been associated with AML and CML.

### 2.3. Gene Ontology

GO analysis was performed using the software package CytoScape 3.8.1 [21] with integrated application ClueGO v2.5.7 [22]. ClueGO analysis was performed using the following parameters and selected options: Ontology/Pathways selected: Biological Process, Cellular Component and Molecular Function Evidence selected: only All_Experimental. Statistical significance cut-off was set at the *p*-value < 0.05 after Bonferroni correction for multiple testing applied automatically by ClueGo software. To enhance biological process discovery with gene ontology analysis, gene networks were constructed by including genes which interact with at least two differentially expressed genes. Biomarkers interacting with at least two differentially expressed genes of interest were obtained from BioGRID database [23,24] using BioGRID R package [25] for R 4.0.2 [12].

## 3. Results

**Figure 1:**
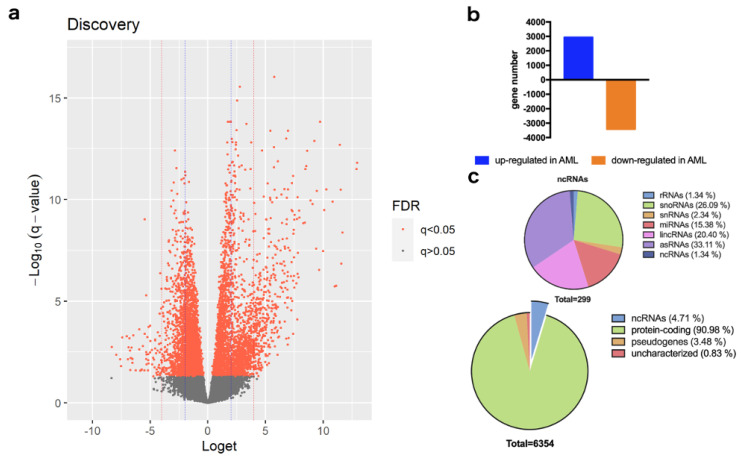
Differentially expressed genes between AML and CML: (**a**) Volcano plot of DEGs. The negative log10 value of the adjusted *q*−values (y−axis) are plotted against the loget value (x−axis). Statistically significant *q*−values are plotted with orange dots. The blue and red vertical intercept lines are set at absolute loget values of 2 and 4, respectively. Note that ORM1 (adjusted *q*−value = 2.15 × 10^−23^) has been omitted for better plot scaling and clarity; (**b**) Of 6354 DEGs, 2935 genes are up-regulated and 3419 genes are down-regulated in AML; (**c**) Classification of DEGs and non-coding RNAs by gene type and function, respectively.

Meta-analysis of global transcriptome data from 5 independent studies (Table 1), using 0.05 *q*-value (*p*-value after FDR correction) cut-off, showed a total of 6354 DEGs between AML and CML, whereby 2935 genes were up-regulated, and 3419 genes were down-regulated in AML in comparison to CML (Figure 1a,b; Table S1). Classification of DEGs by gene type showed that 90.98 % of genes belong to the protein-coding genes, 4.71% to non-coding RNAs (ncRNAs), 3.48% to pseudogenes and 0.83% of genes were uncharacterized (Figure 1c). Among ncRNAs, 1.34% belonged to ribosomal RNAs (rRNAs), 26.09% to small nucleolar RNAs (snoRNAs), 2.34% to small nuclear RNAs (snRNAs), 15.38% to microRNAs (miRNAs), 20.40% to long intergenic non-protein coding RNAs (lincRNAs), 33.11% to antisense RNAs (asRNAs) and 1.34% to other ncRNAs (Figure 1c). Of the first 100 most significant DEGs, we found 5 candidate genes, all up-regulated in AML, that have not yet been associated with AML or CML (Table 2). Functionally, these genes encode for the long intergenic non-protein coding RNA LINC01554 and the pseudogenes PTMAP12, LOC644936, RPS27AP20 and FAM133CP. Finally, to further improve interpretation of DEGs, we performed transcriptome analysis of healthy samples versus AML or CML (Table S2).

### Differentially Expressed Genes between AML and CML belong to Distinct Functional Groups

GO analysis of the top 100 DEGs revealed an association of CML with mRNA processing (number of associated genes: 13/100), spliceosomal complex (14/100), mammalian spliceosomal complex C (6/100) and U2-type spliceosomal complex (10/100), U2-type precatalytic spliceosome (8/100), glycoprotein metabolic process (7/100), reactive oxygen species metabolism (4/100), superoxide anion generation (3/100), specific granules (4/100) and secretory vesicles (6/100), neutrophil migration (4/100), antimicrobial humoral response mediated by antimicrobial peptide (4/100) and defense response to fungi (3/100). AML was associated with the regulation of reactive oxygen species metabolism (4/100) and vesicle localization (3/100) (Table 3). A detailed analysis of spliceosome GO term network showed the high interconnection of pre-RNA splicing processes and suggested that genes and processes associated with the U2-type spliceosome and U6 snRNA may be independent of other spliceosome complexes and snRNAs (Figure 1). Complete results of GO analysis are available in Supplementary Table S3.

## 4. Discussion

Over the past decades genetic and epigenetic factors associated with acute and chronic myeloid leukemia have been well characterized. However, our knowledge of molecular substrates and mechanisms underlying the development and progression of acute myeloid leukemia (AML) and chronic myeloid leukemia (CML) remains limited. Furthermore, the similarities and differences of AML and CML on the transcriptome level have yet to be studied. To our best knowledge, our study is the first to compare transcriptome profiles of AML and CML using modern bioinformatic methodology. In the current study we characterized novel candidate genes whose expression differed between AML and CML. Furthermore, our results provide evidence that AML and CML differ in gene clusters that regulate pre-RNA splicing, reactive oxygen species and glycoprotein metabolism, the endomembrane system, neutrophil migration and antimicrobial immune response.

AML and CML together account for approximately half of all new leukemia cases worldwide [26]. From a cytological perspective, CML is a type of myeloproliferative neoplasm characterized by clonal expansion mainly of granulocytes and their precursors in the peripheral blood and bone marrow. On the other hand, AML results from the clonal expansion of myeloid blasts in the peripheral blood, bone marrow, or other tissues. The spectrum of genetic changes that lead into AML is broad and defines clinical outcome of the disease. Despite the progress in the application of omics technologies in studying myeloid neoplasms and innovations in biotechnology for advanced therapy development, the mortality rate in AML and CML is still high [27,28,29]. Therefore, there is an urgent need for a discovery of new molecular pharmacological targets. In our study, by comparing the transcriptome expression data of AML and CML along with comprehensive literature survey, we identified 5 potential candidate genes whose expression has not yet been associated with AML or CML (Table 2). 

### 4.1. Long Non-Coding RNAs and Pseudogenes

Our results confirmed previous observations implicating a role of non-coding RNAs in AML and CML. Long non-coding RNAs (lncRNAs) are a type of RNA species that do not translate into proteins. By interacting with the DNA, RNA and proteins, lncRNAs are involved in the regulation of gene expression at multiple levels including modulation of chromatin structure and function, DNA methylation, RNA splicing, stability and translation, providing platforms for assembly and guidance of multiple-component complexes and sequestering regulatory molecules [30,31,32,33]. Considering the pathophysiology of AML and CML, it has been shown that lncRNAs regulate various aspects of leukemogenesis and are directly involved in chemoresistance [31,34,35]. Indeed, lncRNAs are considered attractive targets for new therapeutic approaches [36].

Among the first 100 most significant DEGs we characterized lincRNA LINC01554, whose expression has not yet been associated with AML and CML. However, the expression of LINC01554 has been associated with other human cancers. For example, expression of LINC01554 in hepatocellular cancer was lower in comparison to normal tissue, and the reduced expression of LINC01554 was in correlation with the advanced tumor stage, metastasing, tumor recurrence and shorter overall survival [37,38,39]. Similarly, reduced LINC01554 expression and its association with advanced tumor stage and reduced overall survival was characteristic of epithelial ovarian cancer [40]. A role of LINC01554 in tumorigenesis was further corroborated a recent mechanistic study in showing that LINC01554 promotes metastasis of esophageal squamous cell carcinoma [41]. 

Pseudogenes are remnants of functional genes that have lost the ability to encode a functional protein of the original gene. Several lines of evidence suggest that pseudogenes or RNA and protein entities derived from pseudogenes can affect function and expression of the parental as well as unrelated genes and thus are involved in the pathophysiology of human cancer [42]. For hematological malignancies, the expression of BMI1P1 [43], TPTEP1 [44] and VIM2P [45] was decreased in patients with AML. The expression of DUSP5P1, which was increased in AML patients compared to controls, was associated with a higher number of blasts in the bone marrow [46]. Moreover, chromosomal translocations that are characteristic of AML not only change the architecture of protein-coding genes but may also affect neighboring pseudogenes, which in turn may play a role in the pathophysiology of disease. For instance, the 11p15.5 breakpoint region in children with AML contains RDPX1 pseudogene [47] and translocations t(8;21) and t(3;21) span the region of the RUNX1 gene locus with pseudogenes RPL34P3, EZH2P1, RPS20P1, MTND2P1 and MTCO1P1 [48]. Finally, OCT4-PG1 pseudogene interfered with the expression of its parental gene OCT4 in the chemically modified CML cell line K562 resulting in altered expression of ABC transporters, involved in multidrug resistance of CML cells [49].

Our global transcriptome meta-analysis identified 5 novel candidate pseudogenes among the top 100 DEGs whose expression differs between AML and CML (Table 2). PTMAP12 derives from the prothymosin alpha gene, RPS27AP20 from the RPS27A gene encoding the ribosomal protein S27a, FAM133CP from the family with sequence similarity 133 gene member. LOC644936 has a sequence similar to actin-beta gene. Importantly, none of the five novel markers have been detected in healthy individuals from study GSE100026, suggesting their potential involvement in pathogenic oncogene processes. Further studies are needed to confirm the role of the newly characterized pseudogenes in human cancer, including myeloid leukemia.

### 4.2. Pre-RNA Splicing and Processing

**Figure 2:**
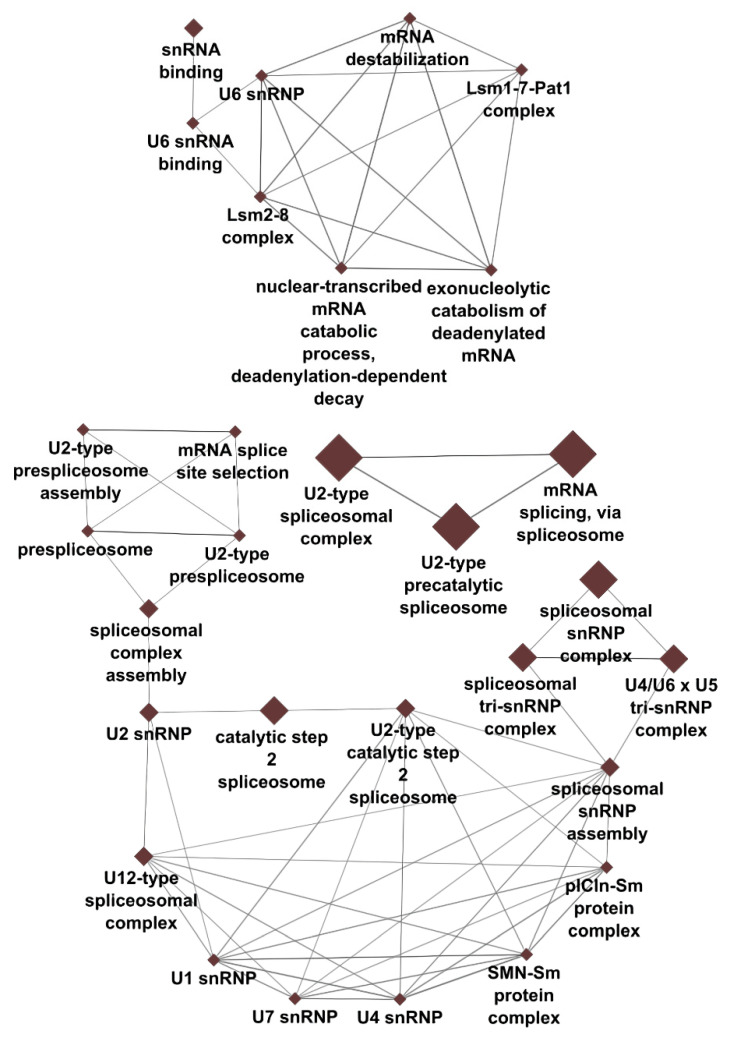
Detailed spliceosome gene ontology term network. Genes and processes related to pre-RNA splicing are significant in CML, but not in AML. U2-type spliceosome and U6 snRNA may be independent of other spliceosome complexes and snRNAs. Node size corresponds to the number of genes associated with the gene ontology term.

Functional analysis revealed that DEGs were enriched in 14 GO terms, with most significant being cellular processes associated with pre-RNA splicing via the U2 spliceosome (Table 3). Mutations in genes encoding splicing factors are frequently found in patients with myelodysplastic syndromes and AML [50,51,52]. Somatic mutations and copy number changes of genes encoding spliceosomal proteins and splicing regulatory factors can affect the expression of tumor-promoting and suppressing genes resulting in hematological malignancies [51,52,53,54]. In addition, altered expression of splicing factors, such as subunit 1 of splicing factor 3 (SF3B1), may also be involved in myelodysplastic syndrome and AML [55]. Our results are in line with these observations and further suggest that the expression of spliceosomal proteins, splicing regulatory factors and snRNAs differ between AML and CML. Our GO term network generated with genes related to our significant spliceosome-related results demonstrates the high interconnection of pre-RNA splicing processes, with U2-type spliceosome- and U6 snRNA-associated genes and processes being potentially independent of other spliceosome complexes and snRNAs (Figure 2). However, additional targeted validation studies are needed to confirm the precise role of the spliceosome in the pathogenesis of AML and CML.

### 4.3. Oxidative Stress

Regulation of cellular redox homeostasis is one of the most important aspects in the pathophysiology of AML and CML. Myeloid leukemias are characterized by high tolerance to elevated levels of reactive oxygen species (ROS) and resistance to oxidative stress, and molecular genetic studies have shown decreased activity of the antioxidant system in leukemia cells. This may be due to somatic mutations and polymorphisms in the promoter and coding regions as well as indirect actions of epigenetic mechanisms, as in the case of peroxiredoxin (PRDX4) in AML, whose expression coincides with histone 3 (H3K23me3) methylation of its promoter [56,57,58]. Nevertheless, it is believed that tolerance to high intracellular ROS concentrations drives leukemogenesis. Here, we show that AML and CML differ in the expression of a set of genes including golgi brefeldin A resistant guanine nucleotide exchange factor 1 (GBF1), membrane glycoprotein CD177, haptoglobin (HP), haptoglobin-related protein (HPR), proteoglycan 3, pro eosinophil major basic protein 2 (PRG3) and apoptosis antagonizing transcription factor (AATF). GBF1 is a Sec7 domain-containing guanine nucleotide exchange factor that activates the ARF family of small GTPases involved in the Golgi biosynthesis, protein trafficking between the endoplasmic reticulum (ER) and the Golgi apparatus and proper processing and sorting of lysosomal cargo (62, 63). Because lysosomes are a source of ROS and enzymes involved in ROS production, it would be reasonable to assume that altered GBF1 expression may interfere with intracellular redox homeostasis. In addition to oxidative phosphorylation in mitochondria and endocytic and phagocytic pathways, secretory pathways involved in innate and humoral immune responses are an important source of ROS in immune cells. CD177 is a glycosylphosphatidylinositol-anchored protein exclusively expressed in a subset of neutrophils [59] and forms a high-affinity complex with serine protease proteinase-3 (PR3). Activation of CD177 by antineutrophil cytoplasmic antibodies against PR3 resulted in degranulation and superoxide production in CD177+PR3+ neutrophils [60], suggesting a role of CD177 in neutrophil activation and ROS production. This was further confirmed in an in vitro functional analysis of subsets of CD177+ and CD177− neutrophils from IBD and AAV patients showing an association between CD177 and increased production of ROS [61,62]. According to the clinical study, patients with Ph-negative chronic myeloproliferative disorders showed and increased expression of CD177 as a result of abnormal proliferation of CD177+ neutrophils in the circulation [63]. Our results further suggest that the expression of CD177 differ between AML and CML. Whether this observation stem from differences in affected cell lineages between AML and CML and reflects intrinsic pathogenicity characteristics of the disease in terms of ROS production warrants further investigation.

Haptoglobin (HP) and haptoglobin-related protein (HPR) are hemoglobin binding plasma proteins. A main function of HP is to bind free hemoglobin to prevent oxidative properties of heme and induce hemoglobin degradation (66). Unlike HP, HPR forms complexes with free hemoglobin that show peroxidase activity and participate in innate immunity (67). A function of HP and HPR in myeloid leukemia is yet to be determined. A previous cDNA microarray study reported increased expression of HPT in cancer than normal blood cells from CML patients (68). Similarly, increased HPT levels have been reported in sera from AML patients (69). Our result confirmed previous observations on abnormal HP expression in AML and CML and further suggest a role of HPR in the pathogenesis of AML and CML.

Of note, our study also indicates that leukemic cells in CML exhibit increased expression of procollagen C-endopeptidase enhancer 2 (PCOLCE2). In an in vitro co-culture model, increased expression of PCOLCE2 in tonsil-derived mesenchymal stem cells was associated with an increased production of ROS in the myelogenous HL-60 cell line [64]. Thus, it would be interesting to assume that de-differentiated leukemic cells, such as blast cells, in CML could affect increased ROS production in surrounding leukemic cells through intercellular communication, ultimately driving the progression of CML from indolent chronic phase to acute phase. 

### 4.4. Protein Glycosylation

The addition of glycan structures or glycosylation is one of the most common modifications of proteins and lipids and thus represents an important pathophysiological aspect of hematological malignancies. Aberrant glycosylation patterns in terms of altered glycan structure and glycosylation sites, which in turn affect cell signaling and communication, cell proliferation and survival, migration and invasion, cellular interaction with the extracellular matrix in bone marrow and endoepithelium and immune modulation have been associated with cancer development, progression and recurrence as well as resistance to chemotherapy [65]. Glycosylation is a complex process that relies on the synergistic action of several enzymes that catalyze the synthesis, modification and binding of carbohydrate moieties to a target molecule. In the present study, we showed that AML and CML differ in glycosylation catalyzed by ALG8 alpha-1,3-glucosyltransferase (ALG8), carbohydrate sulfotransferase 14 (CHST14), exostosin glycosyltransferase 2 (EXT2), protein O-linked mannose N-acetylglucosaminyltransferase 1 (beta 1,2-) (POMGNT1), protein O-mannosyltransferase 2 (POMT2) and transmembrane protein 258 (TMEM258) and 59 (TMEM59). Our knowledge of the function of the aforementioned genes in AML and CML is sparse. There is one study suggesting diminished expression of the transmembrane glycoprotein complex dystroglycan along with altered mRNA expression of enzymes involved in dystroglycan glycosylation, including POMGnT1 and POMT2 in myeloid blasts from AML patients [66].

### 4.5. Extracellular Vesicles and Secretory Granules

Our study suggests that AML and CML differ in the regulation and function of the endomembrane system of cells at the level of genes encoding GBF1, huntingtin (HTT) and spatacsin (SPG1) [67,68,69]. Notably, huntingtin levels are significantly different between AML and CML, but not between healthy controls and AML or CML, implying that it is a non-oncogene marker that may differentiate between AML and CML (see Table S2). Vesicles are membrane structures that in the primarily carry information between cellular membrane organelles including the ER, the Golgi apparatus and mitochondria. Membrane-bound vesicles lysosomes interact with vesicles that derive from one of three pathways: endocytosis, phagocytosis and autophagocytosis. In addition, the ER and the Golgi apparatus are a source of vesicles destined for the plasma membrane or cell microenvironment, enabling intercellular communication. A study by Caivano and co-workers showed higher levels of extracellular vesicles, which also differed in respect to vesicle size and immunophenotype, in peripheral blood in patients with various hematological malignancies, including AML and CML compared to healthy controls [70]. Moreover, in patients affected with AML and CML, total plasma levels of exosomes fractions and EV content were reported to reflect disease stage [71,72,73,74], suggesting their role in the pathogenesis of AML and CML. Growing evidence indicate that in AML and CML extracellular vesicles (EVs), produced by cancer blasts and bone marrow stromal cells modulate the activity of cancer and various non-cancerous cells thereby driving the progression of disease and causing disease relapse and chemoresistance [75,76,77,78,79]. For example, EVs derived from AML cells promoted cancer immune escape by expansion and proliferation of regulatory T lymphocytes and suppression of NKG2D- dependent NK cell cytotoxicity [80,81]. In addition, exosomes released from the bone marrow stromal cells from AML and CML patients had the capacity to modulate microenvironment in the bone marrow that promote growth and migration of leukemia cells [82,83]. The function of GBF1, HTT and SPG11 in the pathophysiology of AML and CML is unknown, although a study of huntingtin interacting protein 1 (HIP1) expression in AML patients suggests that HTT may be associated with overall survival of AML patients via HIP1 [84]. Interestingly, neither of our analysis (AML vs. CML, healthy vs. AML, healthy vs. CML) showed any statistical significance for HIP1 (see Table S2).

In addition to extracellular vesicles, our study suggests that AML and CML also differ in gene expression of neutrophil secondary granule proteins. Granular proteins have been identified as important modulators of neutrophil migration and passage of neutrophils across the endothelium [85,86]. Neutrophils interact with innate and adaptive immune cells and orchestrate immune responses [87,88,89]. Moreover, neutrophil immunity has been recognized as a common mechanism underlying numerous pathological conditions, including autoimmune diseases and cancer [88,90]. Cysteine rich secretory protein 3 (CRISP3), cathelicidin antimicrobial peptide (CAMP), lactoferrin (LTF), olfactomedin 4 (OLFM4) and CD177 have been implicated in the regulation of neutrophil migration, transendothelial migration and antimicrobial immune response. Whether the differences in mRNA expression of genes associated with extracellular vesicles (GBF1, HTT, SPG11) and secretory granules (CRISP3, CAMP, LTF, OLFM4, DC177) between AML and CML reflect differences in the pathogenesis of AML and CML or are simply the result of different myeloid lineages affected by a disease is unknown and is yet to be determined. However, patients with AML and CML may differ in the amount and composition of serum-derived EVs [70].

### 4.6. Migration of Neutrophils

Although observations on the chemotactic ability of neutrophils in CML may be inconsistent (91–93), scientific evidence demonstrates impaired neutrophil migration in CML, which may depend on the stage of myelodysplastic syndrome (92–94). Whether the same applies for AML as well is to the best of our knowledge unknown. Our results showed that AML and CML differ in the expression of GBF1, CD177, OLFM4 and peptidylprolyl isomerase B (PPIB) implicated in the intercellular interactions and chemotaxis regulation to regulate tumor cell migration and invasion [67,85,86,91,92,93,94,95]. Additional studies are needed to evaluate the efficacy of GBF1, CD177, OLFM4 and PPIB as molecular targets for anticancer therapy.

### 4.7. Antimicrobial Immune Response

In addition to the altered neutrophil migration in patients with myeloproliferative disorders, evidence suggests that neutrophils from peripheral blood also manifest to some extent diminished bactericidal and fungicidal activities [96,97]. Moreover, the lower antimicrobial activity of neutrophils may be specific for acute leukemia and the acute phase of chronic leukemia as indicated by LTF immunoreactivity [98]. Our results further suggest that besides LTF AML and CML cells differ in the expression of genes encoding other proteins with antimicrobial activity as part of the innate and humoral immune system, namely, cathelicidin antimicrobial peptide (CAMP), which is cleaved into a potent antimicrobial peptide LL-37, arginase 1 (ARG1), peptidoglycan recognition protein (PGLYRP1) and calcium-binding protein S100A12. The mechanisms of antiviral, antibacterial and antifungal activities of LTF, CAMP/LL-37, ARG1, PGLYRP1 and S100A12 have been well characterized [99,100,101,102,103,104,105]. Recent evidence also indicate that antimicrobial peptides have a role in human cancer, including myeloid malignancies. ARG1 depletes microenvironment of arginine, an amino acid essential for T lymphocyte function. Indeed, expression of ARG1 in purified myeloid-derived suppressor cells suppressed T-cell proliferation in vitro [106]. Thus, higher expression of ARG1 and expansion of myeloid-derived suppressor cells in CML patients may lead to an immunotolerant environment that contributes to CML immune escape [106]. Similarly, suppression of T lymphocyte activity by ARG1 and other proteins of APOE/LILRB4 signaling pathway has been suggested for AML cells [107]. Furthermore, in vitro and in vivo studies on the effects of antimicrobial peptides and vitamin D derivatives on human myeloid and leukemia myeloid cells [108,109] suggests that CML cells may be differentiated to protumorigenic M2 macrophages during disease development and progression to escape inflammation. In contrast, LL-37, ARG1, PGLYRP1 and S100A12 also exhibit antitumor activity affecting tumor cells directly or through the modulation of specific immune and nonimmune stromal cell populations [110,111,112,113,114,115,116]. Taken together, LTF, ARG1, PGLYRP1, S100A12 and CAMP/LL-37 affect the human immune system at various levels by producing both protumorigenic and anticancer effects in a tissue- and cancer-specific manner. Differences in peripheral blood antimicrobial activity between AML and CML have already been suggested. However, whether differences in the expression of LTF, ARG1, PGLYRP1, S100A12, and CAMP /LL-37 between AML and CML are reflected in differences in immunomodulatory effects and disease progression remains to be determined.

## 5. Conclusions

Acute and chronic myeloid leukemia arise from clonal expansion of leukemic myeloid cells into the bone marrow, circulatory system and surrounding tissue. Here, we showed that AML- and CML-specific pathogenesis may rely upon the interplay of gene clusters underlying aberrant pre-RNA splicing, protein glycosylation, redox homeostasis and secretion of immunomodulatory factors that derive from extracellular vesicles and secretory granules, orchestrating neutrophil migration and the innate and humoral immune system, which in turn create a microenvironment for disease progression, relapse and chemoresistance. In addition, we characterized a novel lincRNA and pseudogenes associated with acute and chronic myeloid leukemia representing viable molecular targets for anticancer therapy. However, further studies are needed to validate our findings.

## Figures and Tables

**Table 1:** Data series included in the meta-analysis.

Accession Number	Phenotype	Number of Unique Blood Samples Analyzed	Study Description
GSE52656	AML	22	Leucogene; mutations and gene expression in AML [5,6]
GSE108003	AML	12	gene expression markers for drug sensitivity in AML [7]
GSE108266	AML	6	chromatin landscape and gene expression in different AML subtypes [8]
GSE147524	AML	16	tumor-specific antigens in AML [9]
GSE100026	CML	10	gene expression in CML [10]
GSE100026	Healthy	5	gene expression in CML [10]

**Table 2:** Novel candidate genes associated with AML and CML.

Symbol	Gene Name	*q*-Value
*PTMAP12*	prothymosin alpha pseudogene 12	9.20 × 10^−17^
*LOC644936*	actin beta pseudogene	4.07 × 10^−14^
*RPS27AP20*	RPS27A pseudogene 20	2.64 × 10^−13^
*FAM133CP*	family with sequence similarity 133, member A pseudogene	3.83 × 10^−^^13^
*LINC01554*	long intergenic non-protein coding RNA 1554	2.53 × 10^−12^

**Table 3:** Gene ontology of differentially expressed genes between AML and CML.

Go Annotation	Associated Genes *	*p*-Value	Specificity
Spliceosomal complex	*CWC22, IK, ISY1, LSM6, PNN, PRPF3, PRPF38A, PRPF8, RBMX2, RNF113A, SLU7, SYF2, ZCRB1, ZRSR2*	8.33 × 10^−9^	CML
U2-type spliceosomal complex	*CWC22, IK, ISY1, LSM6, PRPF3, PRPF38A, PRPF8, RBMX2, RNF113A, SYF2*	3.29 × 10^−7^	CML
U2-type precatalytic spliceosome	*CWC22, IK, LSM6, PRPF3, PRPF38A, PRPF8, RBMX2, RNF113A*	1.45 × 10^−6^	CML
mRNA processing	*AKAP8L, CWC22, IK, ISY1, LSM6, PRPF3, PRPF38A, PRPF8, RBMX2, RNF113A, SLU7, SYF2, ZRSR2*	3.57 × 10^−5^	CML
Specific granule	*CAMP, CRISP3, LTF, OLFM4*	2.33 × 10^−4^	CML
Catalytic step 2 spliceosome	*CWC22, ISY1, PNN, PRPF8, SLU7, SYF2*	3.92 × 10^−3^	CML
Secretory granule	*CA4, CAMP, CD177, CRISP3, LTF, OLFM4*	4.55 × 10^−3^	CML
Antimicrobial humoral immune response mediated by antimicrobial peptide	*CAMP, LTF, PGLYRP1, S100A12*	1.34 × 10^−2^	CML
Reactive oxygen species metabolic process	*AATF, CD177, GBF1, HP, HPR, PRG3*	1.39 × 10^−2^	CML
Glycoprotein metabolic process	*ALG8, CHST14, EXT2, POMGNT1, POMT2, TMEM258, TMEM59*	1.44 × 10^−2^	AML
Superoxide anion generation	*AATF, CD177, PRG3*	1.53 × 10^−2^	CML
Neutrophil migration	*CD177, GBF1, OLFM4, PPIB*	1.57 × 10^−2^	CML
Defense response to fungus	*ARG1, LTF, S100A12*	1.58 × 10^−2^	CML
Regulation of reactive oxygen species metabolic process	*AATF, CD177, HP, HPR*	1.65 × 10^−2^	CML
Establishment of vesicle localization	*GBF1, HTT, SPG11*	1.99 × 10^−2^	AML

* As per common annotation of gene names, gene symbols are written in italics.

## Data Availability

Data is contained within the article or supplementary material and is available upon request.

## Supplementary Materials

The following are available online at https://www.mdpi.com/article/10.3390/cancers14194681/s1, Table S1: Differentially expressed genes between AML and CML; Table S2: Differentially expressed genes between healthy samples and AML/CML. Table S3: Results of GO analysis.
